# Cloning, Expression, and Functional Analysis of the MYB Transcription Factor *SlMYB86-like* in Tomato

**DOI:** 10.3390/plants13040488

**Published:** 2024-02-08

**Authors:** Na Chen, Wenwen Zhan, Qin Shao, Liangliang Liu, Qineng Lu, Weihai Yang, Zhiqun Que

**Affiliations:** 1 College of Life Science and Resources and Environment, Yichun University, Yichun 336000, China; shaoqin2013@126.com (Q.S.); 190030@jxycu.edu.cn (L.L.); qinenglu@sina.com (Q.L.); seayang2004@126.com (W.Y.); zhiqunque@163.com (Z.Q.); 2 Guangzhou Resuce Agricultural Science and Technology Co., Ltd., Guangzhou 510642, China; 19318872285@163.com

**Keywords:** tomato bacterial wilt, MYB transcription factor, gene cloning, expression analysis, virus-induced gene silencing (VIGS)

## Abstract

MYB transcription factors (TFs) have been shown to play a key role in plant growth and development and are in response to various types of biotic and abiotic stress. Here, we clarified the structure, expression patterns, and function of a *MYB* TF, *SlMYB86-like* (Solyc06g071690) in tomato using an inbred tomato line exhibiting high resistance to bacterial wilt (Hm 2-2 (R)) and one susceptible line (BY 1-2 (S)). The full-length cDNA sequence of this gene was 1226 bp, and the open reading frame was 966 bp, which encoded 321 amino acids; its relative molecular weight was 37.05055 kDa; its theoretical isoelectric point was 7.22; it was a hydrophilic nonsecreted protein; and it had no transmembrane structures. The protein also contains a highly conserved MYB DNA-binding domain and was predicted to be localized to the nucleus. Phylogenetic analysis revealed that SlMYB86-like is closely related to SpMYB86-like in *Solanum pennellii* and clustered with other members of the family *Solanaceae*. Quantitative real-time PCR (qRT-PCR) analysis revealed that the expression of the *SlMYB86-like* gene was tissue specific and could be induced by *Ralstonia solanacearum*, salicylic acid, and jasmonic acid. The results of virus-induced gene silencing (VIGS) revealed that *SlMYB86-like* silencing decreased the resistance of tomato plants to bacterial wilt, suggesting that it positively regulates the resistance of tomatoes to bacterial wilt. Overall, these findings indicate that *SlMYB86-like* plays a key role in regulating the resistance of tomatoes to bacterial wilt.

## 1. Introduction

The tomato (*Solanum lycopersium*) is an economically important vegetable crop with a high nutritional value. The cultivation area of solanaceous vegetable crops, including pepper, tomato, and eggplant, and the production of these crops has increased extensively in recent years. Bacterial wilt disease, a bacterial soil-borne disease caused by *Ralstonia solanacearum* that has been referred to as “plant cancer,” has induced major economic losses of solanaceous crops and poses a major threat to the future growth of the solanaceous crop industry [1,2]. *R. solanacearum* can invade wounds at the base of the plant stem or root system from the soil and multiply in the vascular bundle after invasion, which induces damage to the vascular bundle; the subsequent lack of a normal water supply can lead to the wilting of the stem and leaves and eventually plant death [3,4]. *R. solanacearum* has been documented to infect more than 200 plant species in more than 50 families [5]. The breeding of tomato varieties with improved disease resistance is a critically important approach for the control of tomato bacterial wilt, and analysis of the molecular mechanisms underlying the resistance of tomato plants to bacterial wilt is key for achieving further increases in production.

Plants are exposed to various types of stress, such as low temperature, salt, strong light, drought, and pathogen stress during their growth and development; the expression of various genes underlies the ability of plants to cope with the deleterious effects of stress on growth and development [6]. Transcription factors (TFs) are regulatory proteins with special structures that can regulate the expression of genes. They are capable of binding to specific factors upstream of the 5′ end of genes, which enhances or inhibits gene expression [7]. *MYB* genes comprise one of the largest TF families in plants; *MYB* genes are ubiquitous in most plants and play a role in plant growth, development, and stress responses [8]. The N-terminal of *MYB* TFs contains a highly conserved DNA-binding domain, and the C-terminal contains a transcriptionally active domain. The DNA-binding domain of this TF comprises approximately 51–52 highly conserved amino acid residues (R), and *MYBs* can be divided into four subfamilies according to differences in the numbers of various structures: 1R-MYB (MYB-related), R2R3-MYB, R1R2R3-MYB (3R-MYB), and 4R-MYB. MYB TFs in the R2R3-MYB subfamily are the most common [9,10,11]. *MYB* genes were first isolated from *Zea mays* [12]; various *MYB* genes have been identified since the sequencing of the whole genomes of various plants, especially model plants. A total of 244, 199, 192, 492, and 127 *MYB* TFs have been identified in *Glycine max*, *Arabidopsis thaliana*, *Populus*, *Gossypium hirsutum*, and *S. lycopersicum*, respectively [13,14,15,16,17]. Previous studies have shown that *MYB* TFs play a key role in the growth and development of plants and abiotic stress responses [18,19,20]. An increasing number of studies have focused on clarifying the roles of *MYB* TFs involved in regulating the resistance of plants to biological stress. For example, the *OsJAMyb* gene in rice is expressed in the root, stem, leaf, sheath, immature panicle, and flowering panicle tissue. The expression of *OsJAMyb* is significantly higher in resistant rice varieties than in susceptible rice varieties following inoculation with *Magnaporthe oryzae*, and *OsJAMyb* overexpression increases the resistance of rice plants to *M. oryzae* and leads to decreases in the number of disease spots [21]. Jiang et al. [22] indicated that *MYB* TF, *VqMYB154*, from *Vitis quinquangularis* accession Danfeng-2 promotes the biosynthesis and accumulation of stilbenes and enhances resistance to *Pseudomonas syringae* in grapevine. *GhMYB36* from *Gossypium hirsutum* has been shown to confer *Arabidopsis* and cotton with resistance to *Verticilium* wilt. Further studies have shown that *GhMYB36* is a transcription factor that enhances *Verticillium* wilt resistance in *Arabidopsis* and cotton by enhancing *PR1* expression [23]. Overexpression of the TF gene *CaMYB78* has been shown to increase the resistance of the chickpea to *Fusarium oxysporum* [24]. The two *MYB* genes *CsMYB41* and *CsMYB63* might play a key role in mediating the response of citrus plants to *Xanthomonas citri* subsp. *citri* (*Xcc*) [25]. Shan et al. [26] showed that the overexpression of *TaMYB86* significantly enhances resistance to *Bipolaris sorokiniana* in transgenic wheat lines and that *TaMYB86* plays a positive role in the defense response to *B. sorokiniana*. Gu et al. [27] suggested that *MdMYB73* from “Royal Gala” apples might enhance resistance to *Botryosphaeria dothidea* via the salicylic acid (SA) pathway. Zhu et al. [28] showed that the *MYB* TF, *TaMYB29*, from wheat could be significantly induced by SA, abscisic acid (ABA), jasmonic acid (JA), ethylene (ET), and *Puccinia striiformis* f. sp. *tritici* (*Pst*). Further studies confirmed that *TaMYB29* positively regulates the defense response against stripe rust in wheat via SA signaling-pathway-induced cell death. Zhu et al. [29] showed that the novel R2R3-type *MYB* TF *GhODO1* from cotton (*Gossypium hirsutum*) promotes the resistance of cotton to *Verticillium dahliae* through lignin biosynthesis and the JA signaling pathway.

An increasing number of studies have examined the roles of *MYB* TFs in the growth and development of tomato plants and responses to biotic and abiotic stress [30,31,32,33]. However, few studies have examined the roles of *MYB* TFs in the bacterial wilt resistance of the tomato. Here, we identified the *MYB* TF *SlMYB86-like* (Solyc06g071690), which shows significant differences in expression, using two inbred tomato lines, one with high resistance to bacterial wilt (Hm 2-2 (R)) and one with high susceptibility to bacterial wilt (BY 1-2 (S)). The *SlMYB86-like* gene was cloned using a reverse transcription polymerase chain reaction, and bioinformatics analysis was conducted. The tissue specificity of the expression of *SlMYB86-like* and its expression patterns under different exogenous hormones and bacterial wilt stress were analyzed using quantitative real-time PCR (qRT-PCR). Virus-induced gene silencing (VIGS) was used to clarify the function of *SlMYB86-like* in the regulation of tomato bacterial wilt resistance. The aim of this study was to provide insights into the mechanisms by which MYB TFs mediate interactions between the tomato and bacterial wilt.

## 2. Results

### 2.1. Cloning of Tomato SlMYB86-like and Gene Structure Analysis

**Figure 1:**
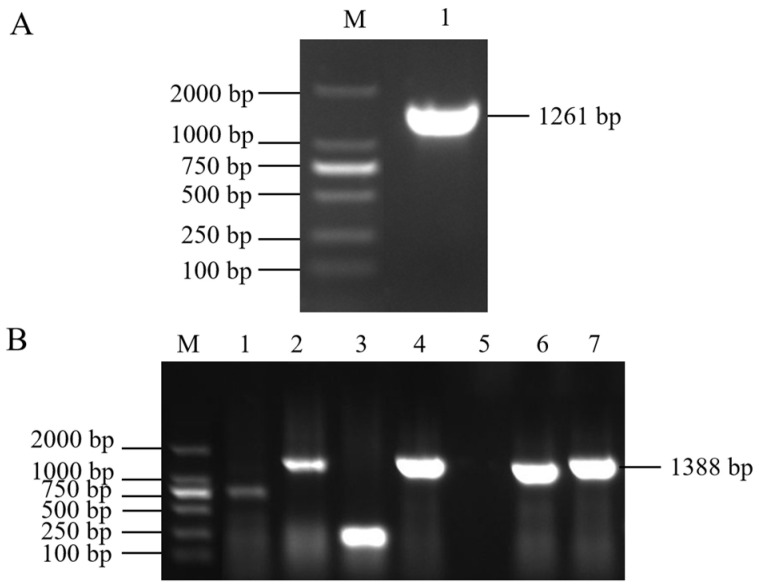
Agarose gel electrophoresis picture. (**A**) Cloned cDNA fragment of *SlMYB86-like* gene. M: Mark DL 2000, 1: *SlMYB86-like* gene. (**B**) Vector construction. M: Mark DL 2000; 2, 4, 6, 7: Positive clone; 1, 3, 5: Negative clone.

**Figure 2:**
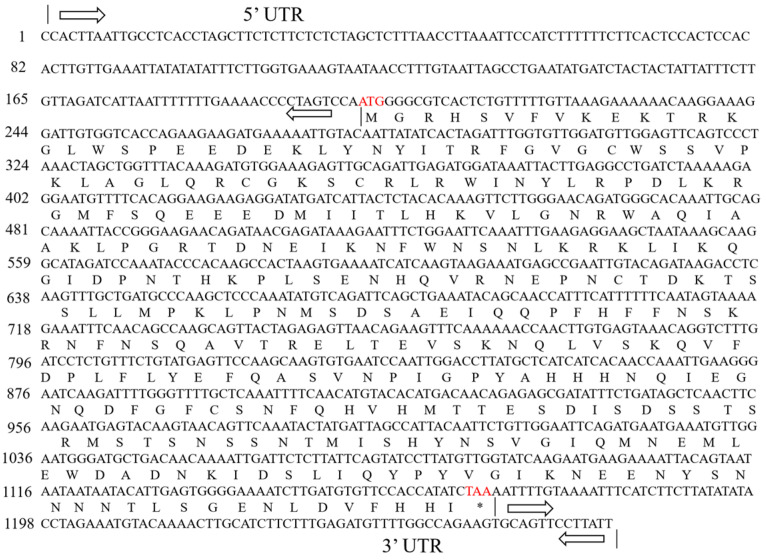
cDNA sequence of *SlMYB86-like* and predicted ORF. ATG: Start codon; TAA: Stop codon; *: End; arrows: Untranslated region.

**Figure 3:**
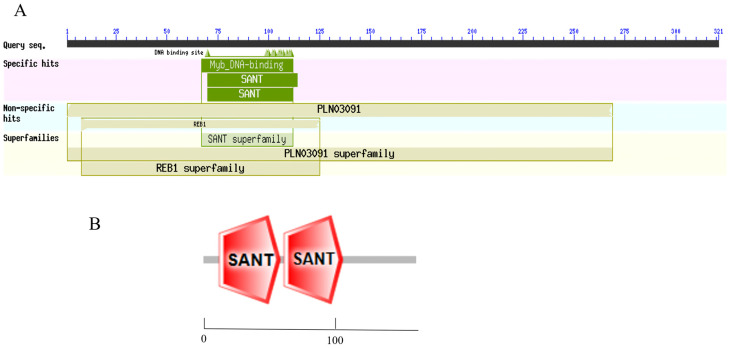
Prediction of SlMYB86-like protein domain. (**A**) Conservative domain of SlMYB86-like protein. (**B**) Prediction analysis results of SlMYB86-like protein structure.

**Figure 4:**
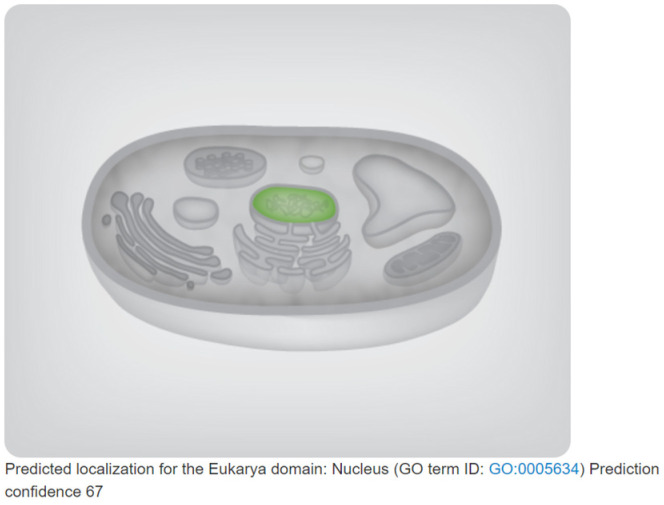
Subcellular localization prediction of *SlMYB86-like*.

Electrophoretic bands of 1261 bp were cloned via PCR from BY 1-2 (S) tomato seedlings (Figure 1A); the size of the bands was consistent with the expected size of the target gene fragment. The PCR-amplified products were recovered, purified, and cloned into the pMD19-T vector (Figure 1B). The sequencing results revealed that the *SlMYB86-like* gene was 1261 bp, with an open reading frame (ORF) of 966 bp and encoded a protein with 321 amino acids (starting at 201 bp and ending at 1166 bp) (Figure 2). A BLAST search of the cDNA sequence of the *SlMYB86-like* gene against the tomato genome (ITAG release 4.0) was performed and assigned the Gene Accession number Solyc06g071690. A conserved domain analysis revealed that SlMYB86-like contains the MYB DNA-binding domain, as well as the PLN03091, REB1, and SANT structure domains (Figure 3A). Furthermore, SMART online program prediction showed that the SlMYB86-like protein had two typical SANT-MYB domains located at amino acids 13–63 and 66–114 (Figure 3B). A PredictProtein online program prediction showed that *SlMYB86-like* was localized to the nucleus (Figure 4).

**Figure 5:**
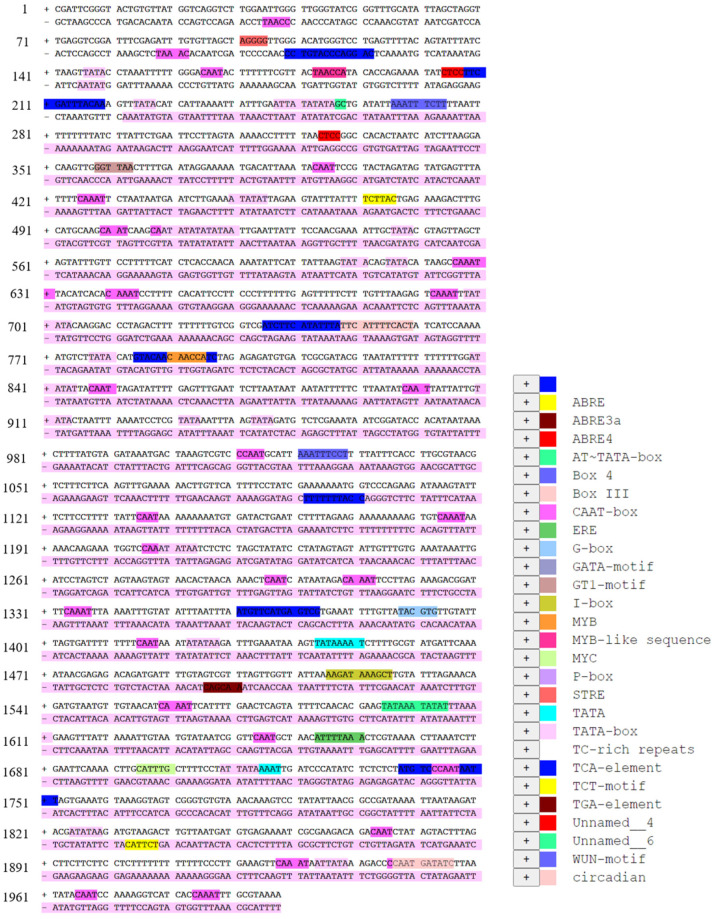
The *cis*-acting element motif prediction of *SlMYB86-like* promoter.

A 2000-bp region of the *SlMYB86-like* promoter was downloaded from the tomato genome, and the *cis*-acting element motifs of the promoter were predicted using the PlantCARE online program (Figure 5, Table S1). Based on these analyses, we identified two MYB core-binding sites (TAACCA). The promoter sequence also contained the core *cis*-acting elements TATA and CAAT, a *cis*-acting element involved in ABA responsiveness, several light-response *cis*-acting elements, a gibberellin-responsive element, a *cis*-acting element involved in defense and stress responses, a *cis*-acting element involved in SA responsiveness, an auxin-responsive element, and a wound-responsive element.

### 2.2. Bioinformatics Analysis of the Tomato SlMYB86-like Gene

**Figure 6:**
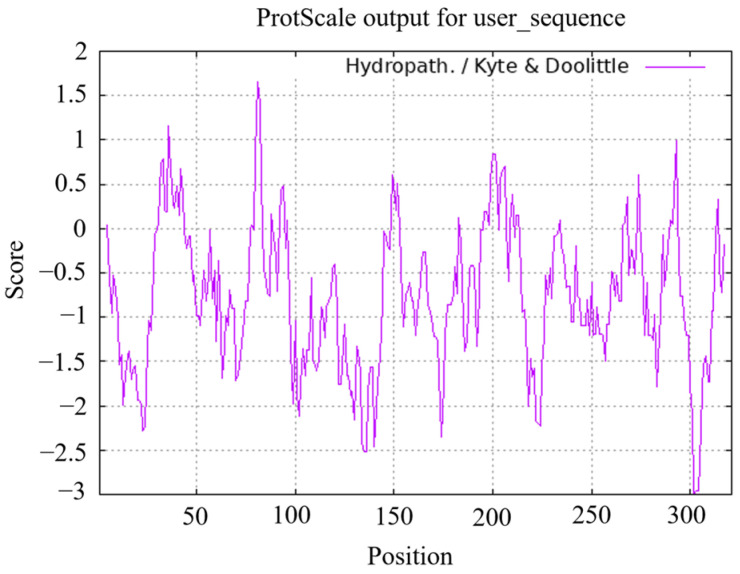
Hydrophilic prediction of SlMYB86-like protein.

**Figure 7:**
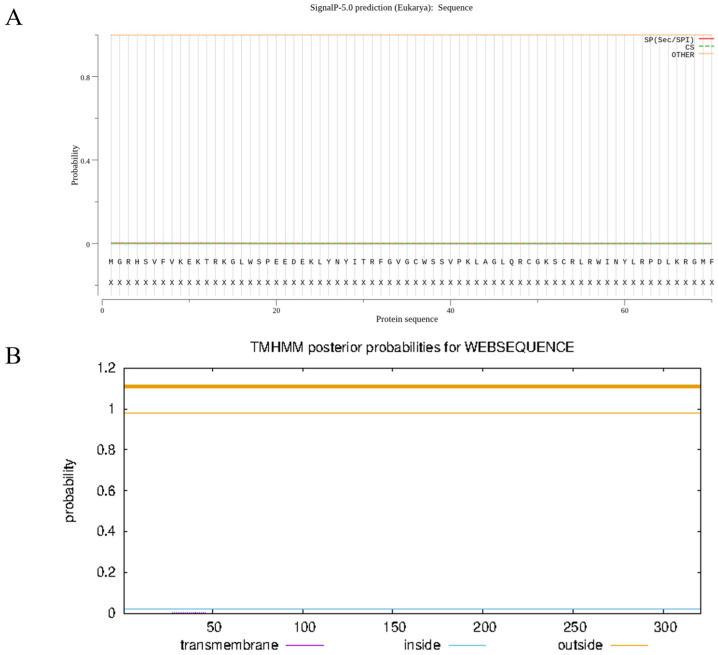
Signal peptide and transmembrane structure prediction of SlMYB86-like protein. (**A**) Signal peptide prediction of SlMYB86-like protein. (**B**) Transmembrane structure prediction of SlMYB86-like protein.

ProtParam analysis revealed that the molecular formula of the protein was C_1626_H_2511_N_467_O_498_S_15_, the molecular weight of the protein was 37.05055 kDa, the theoretical isoelectric point was 7.22, the total number of negatively charged residues (Asp + Glu) was 36, and the total number of positively charged residues (Arg + Lys) was 36. The aliphatic index was 67.38, the instability index was 47.44, and the grand average of hydropathicity was −0.754, indicating that the protein was hydrophilic (Figure 6). In sum, the protein is a weakly alkaline and unstable hydrophilic protein. The SignalP 6.0 and TMHMM Server v.2.0 online programs were used to analyze the signal peptides and transmembrane domains of tomato SlMYB86-like protein, respectively. SlMYB86-like did not contain signaling peptides, indicating that it is a nonsecretory protein; it also did not contain transmembrane structures (Figure 7).

**Figure 8:**
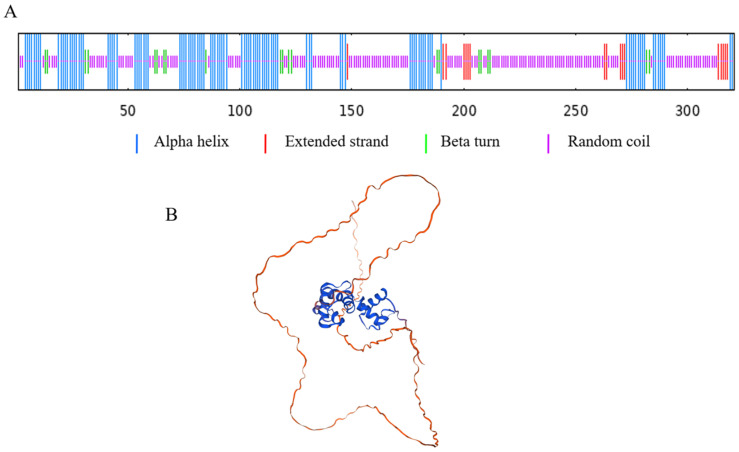
Secondary structure and tertiary structure prediction of SlMYB86-like protein. (**A**) The secondary structure prediction of SlMYB86-like protein. (**B**) The tertiary structure prediction of SlMYB86-like protein.

The SOPMA program was used to analyze the secondary structure of SlMYB86-like. The proportion of random coils and alpha helices was 55.14% and 32.71%, respectively. The proportion of extended strands was 5.30%, and the proportion of beta turns was 6.85% (Figure 8A). The secondary structure of SlMYB86-like protein was, thus, mainly composed of random coils and alpha helices, and the proportion of extended strands and beta turns was relatively low. The SWISS-MODEL online program was used to analyze the three-dimensional structure of SlMYB86-like in the tomato; random coils and alpha helices were the main secondary structures comprising SlMYB86-like (Figure 8B), and these findings were consistent with the secondary structure predictions.

**Figure 9:**
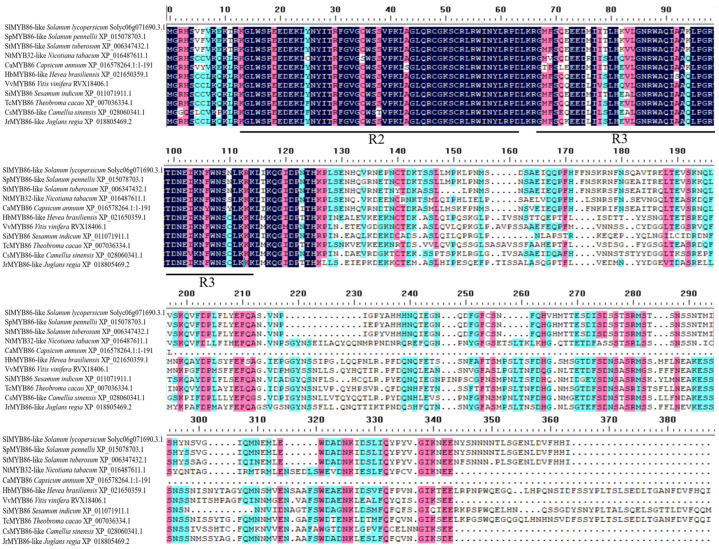
Multiple alignments of homologous protein for the SlMYB86-like in tomato and other species.

**Figure 10:**
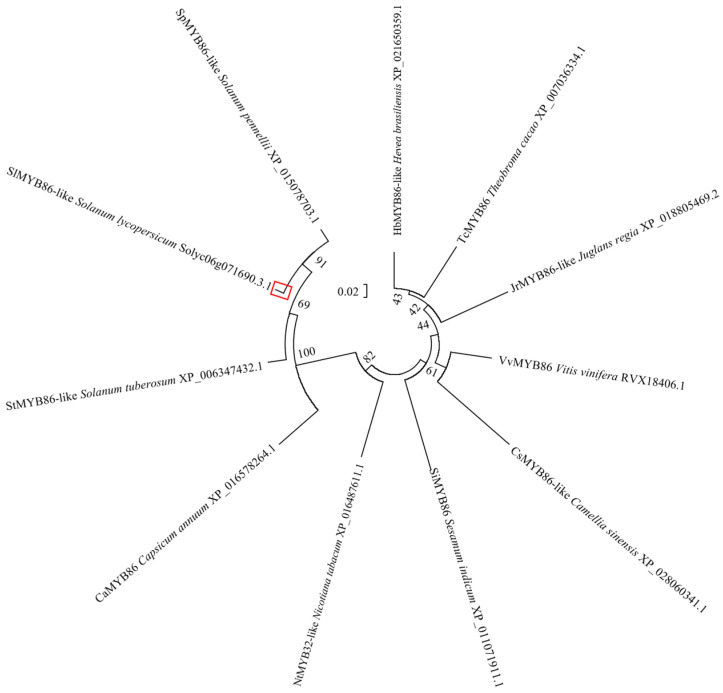
Phylogenetic tree of SlMYB86-like homologous protein. The red box: The SlMYB86-like protein.

The protein sequences of 10 species (e.g., *Solanum pennellii*, *Solanum tuberosum*, *Capsicum annuum*, and *Nicotiana tabacum*) with high homology to SlMYB86-like in tomato were obtained via a BLAST search against the National Center for Biotechnology Information (NCBI) database. The 11 SlMYB86-like proteins all contained an R2 and R3 MYB domain, indicating that they were all R2R3-MYB TFs (Figure 9). Phylogenetic trees were constructed using MEGA6.0 software, and SlMYB86-like (tomato) was the most closely related to SpMYB86-like (*S. pennellii*) and StMYB86-like (*S. tuberosum*) and most distantly related to the homologous proteins in *Theobroma cacao* and *Hevea brasiliensis* (Figure 10).

### 2.3. Analysis of the Tissue-Specific Expression Patterns of SlMYB86-like in Tomato

**Figure 11:**
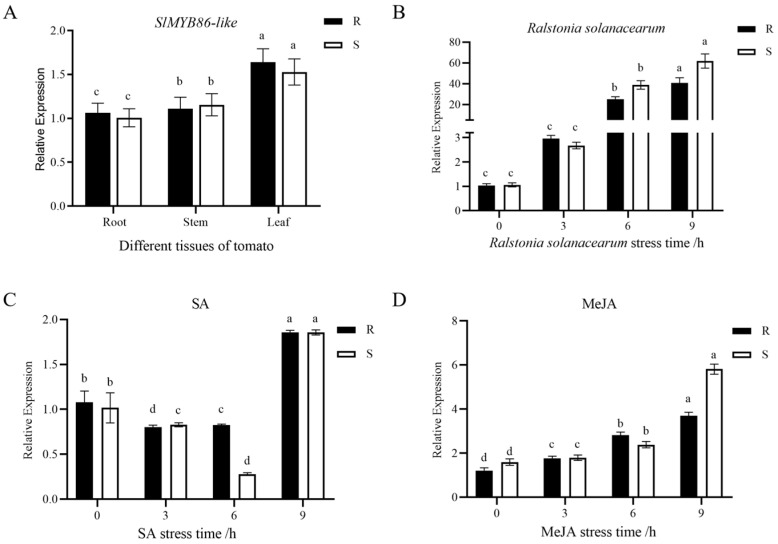
Expression analysis of tomato *SlMYB86-like* gene. (**A**) Expression of *SlMYB86-like* gene in different tomato tissues. (**B**) Expression of *SlMYB86-like* gene under stress of *Ralstonia solanacearum*. (**C**) Expression of *SlMYB86-like* gene under exogenous hormone SA treatment. (**D**) Expression of *SlMYB86-like* gene under exogenous hormone MeJA treatment. *n* = 3, the different lowercase letters represent significant differences (*p* < 0.05).

The expression levels of *SlMYB86-like* in different tissues (root, stem, and leaf) in two tomato inbred lines were analyzed by qRT-PCR using the *Actin* gene as a reference. In both Hm 2-2 (R) and BY 1-2 (S), the expression of *SlMYB86-like* was highest in the leaves, followed by the stems and roots (Figure 11A). The expression of *SlMYB86-like* in tomato was tissue specific.

### 2.4. Analysis of SlMYB86-like Gene Expression under Different Types of Stress in Tomato

The roots of two tomato inbred lines at the five-leaf stage were inoculated with *R. solanacearum* by the root-breaking method, and tomato leaves were sampled at 0, 3, 6, and 9 h for qRT-PCR analysis. The results (Figure 11B) revealed that the expression of *SlMYB86-like* in Hm 2-2 (R) and BY 1-2 (S) increased following inoculation with *R. solanacearum*, suggesting that *R. solanacearum* could induce the expression of *SlMYB86-like*. The expression level of this gene increased with inoculation time in the two inbred tomato lines. Hm 2-2 (R) and BY 1-2 (S) were sampled at 0, 3, 6, and 9 h for qRT-PCR analysis following treatment with the exogenous hormones SA and methyl jasmonate (MeJA). Following SA treatment, the expression of *SlMYB86-like* in Hm 2-2 (R) and BY 1-2 (S) first decreased and then increased. Following MeJA treatment, the expression of *SlMYB86-like* increased in both resistant and susceptible materials. These results indicate that *SlMYB86-like* might play a key role in regulating the resistance of the tomato to bacterial wilt. *SlMYB86-like* might be involved in the response of the tomato to bacterial wilt by regulating the SA and JA pathways.

### 2.5. Functional Analysis of the Role of SlMYB86-like in Regulating the Resistance of Tomato to Bacterial Wilt

**Figure 12:**
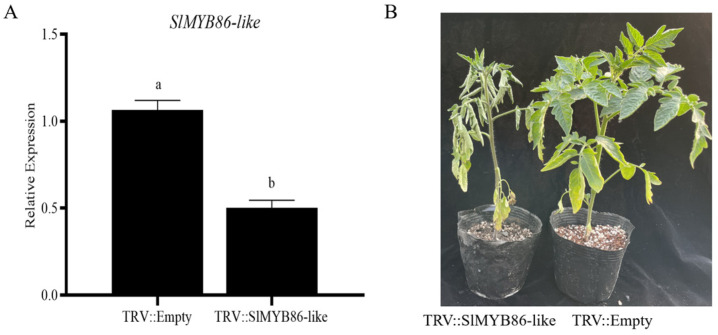
*SlMYB86-like* positively regulates tomato resistance to bacterial wilt. (**A**) *SlMYB86-like* relative expression in seedlings with the *SlMYB86-like* silencing mediated by TRV. *n* = 3, the different lowercase letters represent significant differences (*p* < 0.05). (**B**) Phenotypic characterization of tomato leaf with *SlMYB86-like* silencing mediated by TRV. TRV::Empty: Control group, *trans*-infected with empty virus vector. TRV::SlMYB86-like: Experimental group, *trans*-infected with *SlMYB86-like* recombinant virus vector.

Virus-induced gene silencing (VIGS) technology was used to silence *SlMYB86-like*, and the regulatory effects of this gene on bacterial wilt resistance were analyzed. After silencing *SlMYB86-like* in Hm 2-2 ^®^, the expression level of *SlMYB86-like* was lower in Hm 2-2 (R) than in TRV::Empty plants (Figure 12A), suggesting that *SlMYB86-like* was silenced following TRV-mediated *SlMYB86-like* inoculation. TRV::SlMYB86-like plants and TRV::Empty plants were also inoculated with *R. solanacearum*; no significant changes in the leaves of the plants inoculated with TRV::Empty were observed, and pronounced wilting was observed in the leaves of seedlings inoculated with TRV::SlMYB86-like (Figure 12B). These findings indicate that the silencing of *SlMYB86-like* decreased the resistance of seedlings to bacterial wilt and positively regulated bacterial wilt resistance.

## 3. Discussion

Tomato is one of the most economically important vegetable crops worldwide; it is vulnerable to bacterial wilt during its growth and development, which seriously affects its yield and quality. Identifying genes that regulate resistance to bacterial wilt in the tomato and analyzing their molecular regulatory mechanism can yield excellent genetic resources for the breeding of new tomato varieties with high yield, high quality, and strong resistance through gene editing and molecular marker-assisted breeding. Previous studies have confirmed that plant MYB TFs play a key role in regulating the responses of plants to pathogens; however, the precise role of MYB TFs in tomato bacterial wilt has not yet been reported. We analyzed the expression patterns of *SlMYB86-like* and preliminarily identified its functions to clarify the role of MYB TFs in regulating the response of the tomato to bacterial wilt; our findings have important practical implications. The MYB TF *SlMYB86-like* was cloned from a tomato, and a bioinformatics analysis was performed. An analysis of the gene structure revealed that SlMYB86-like contains an R2 and R3 MYB domain, indicating that it is an R2R3-MYB TF. SMART online program prediction revealed that the SlMYB86-like protein had two typical SANT-MYB domains; this was basically consistent with the results of previous studies of *Gossypium barbadense* [34] and *Macadamia integrifolia* [35]. Dai et al. [36] reported that the cotton *GhMYB6* gene had only one highly conserved SANT domain, indicating that it might belong to a different subfamily. *SlMYB86-like* was predicted to be localized to the nucleus, which was consistent with the results of Luo et al. [37] and Ma et al. [38], as well as with the structural characteristics of TFs. Analysis of the SlMYB86-like protein revealed that it is hydrophilic, which is consistent with the results of Hu et al. [39]. Secondary structure prediction indicated that SlMYB86-like is mainly composed of random coils. These findings were basically consistent with the results of previous studies of kenaf (*Hibiscus cannabinus* L.) [40] and grape hyacinth (*Muscari armeniacum*) [41].

Previous studies have shown that MYB TFs are involved in plant growth and development and responses to biotic and abiotic stress; MYB TFs are also involved in the response of the tomato to various types of biotic stress. For example, Li et al. [42] found that *SlMYB28*, which encodes an R2R3-MYB transcription factor, is a negative regulator of the response to tomato yellow leaf curl virus (TYLCV) infection in the tomato. Liu et al. [43] knocked out the *SlMYBS2* gene in the tomato using CRISPR/Cas9 technology, and the number of necrotic cells, the severity of lesions, and the disease index were higher, and the resistance to Phytophthora infestation was lower in transgenic plants than in wild-type plants. Yin et al. [44] showed that *SlMYB1* is an important multifunctional TF that can regulate the resistance of the tomato to *Botrytis cinerea*. Shan et al. [26] showed that overexpression of *TaMYB86* significantly enhances resistance to *B. sorokiniana* in transgenic wheat lines and that *TaMYB86* plays a positive role in the defense response to *B. sorokiniana*. The results of this study revealed that silencing the *SlMYB86-like* gene reduced the resistance of the tomato to bacterial wilt, suggesting that *SlMYB86-like* positively regulates the resistance of the tomato to bacterial wilt. However, the molecular mechanism underlying its regulatory effects on bacterial wilt resistance in the tomato remains unclear.

Plant stress responses are often related to hormone signal transduction pathways, and MYB TFs are some of the most important proteins in complex plant hormone signaling networks. Plant hormones, especially MeJA and SA, play a key role in regulating the responses of plants to pathogens. MYB TFs play a role in the responses of plants to biotic stress through plant hormones; for example, *AtMYB30* in *Arabidopsis* has been shown to promote the accumulation of SA in plants, which induces the hypersensitive response (HR). *AtMYB30* overexpression in *Arabidopsis* and tobacco leads to the acceleration and enhancement of HR and enhances resistance to *Xanthomonas campestris* pv. *campestris* (*Xcc*). By contrast, inhibition of *AtMYB30* expression impedes the HR and reduces the resistance of *Arabidopsis* to *Xcc* [45]. *SlMYB75* overexpression in tomato plants increases the content of JA, enhances peroxidase (POD) and superoxide dismutase (SOD) activity, and promotes the JA-mediated signaling pathway to clear *B. cinerea* infection, suggesting that *SlMYB75* could enhance the resistance of the tomato to *B. cinerea* [46]. Hawku et al. [47] showed that *TaMYB391* acts as a positive regulator of HR-associated cell death and promotes the resistance of wheat to stripe rust by altering the expression of pathogenesis-related genes, possibly through the SA signaling pathway. We found that the expression of the *SlMYB86-like* gene in the tomato could be significantly induced by *R. solanacearum*, MeJA, and SA treatments, suggesting that *SlMYB86-like* is involved in regulating tomato bacterial wilt resistance and hormone stress responses. This gene might play a role in the response of the tomato to *R. solanacearum* by regulating the MeJA and SA signaling pathways or the MeJA and SA synthesis pathways; however, additional studies are needed to clarify the specific mechanisms.

## 4. Materials and Methods

### 4.1. Plant Materials

Two inbred tomato lines, one with high resistance to bacterial wilt (Hm 2-2 (R)) and one highly susceptible to bacterial wilt (BY 1-2 (S)), were provided by the Key Laboratory of Crop Breeding in South Zhejiang Province, China. The pathogen *R. solanacearum* and VIGS vector pTRV1 and pTRV2 strains were all stored in the Key Laboratory of Crop Growth and Development Regulation of Jiangxi Province, China. Healthy tomato seeds were sown in a pot containing a 3:1 peat–perlite mixture (*v*/*v*). The seeds were grown in a climate-controlled chamber with a daytime temperature of 28–30 °C, nighttime temperature of 15–17 °C, 14/10 h light–dark photoperiod, and relative humidity of 50%. The roots of two inbred healthy tomato seedlings at the five-leaf stage with consistent growth were cultured in a bacterial suspension of 1 × 10^8^ colony-forming units (CFU/mL) using the root-breaking method. Seedlings at the five-leaf stage were sprayed on leaves with 0.2 mmol/L SA solution for SA treatment and 1.5 mmol/L MeJA solution for MeJA treatment. Control plants were inoculated with sterile water. Samples of the top leaf to the third leaf were collected after treatment for 0, 3, 6, and 9 h. There were 15 seedlings in each treatment, and three replications of each experiment were performed. All samples were frozen in liquid nitrogen and stored at −80 °C. The roots, stems, and leaves of five-leaf seedlings with vigorous growth were frozen in liquid nitrogen and stored at −80 °C.

### 4.2. Methods

#### 4.2.1. Extraction of Total RNA and cDNA Synthesis

Total RNA was extracted from BY 1-2 (S) seedlings at the five-leaf stage using the HiPure Total RNA Mini Kit (Magen, Guangzhou, China). First-strand cDNA was synthesized using the HiScript II 1st Stand cDNA Synthesis Kit (+gDNA wiper) (Vazyme, Nanjing, China) and then stored at −20 °C.

#### 4.2.2. Cloning of the Tomato *SlMYB86-like* Gene

To obtain full-length cDNA of *SlMYB86-like*, Primer Premier 5 software was used to design a pair of specific primers (*SlMYB86-like* F/R: CCACTTAATTGCCTCACCTA GC/AATAAGGAACTGCACTTCTGGC). cDNA from the leaves with a high susceptibility to bacterial wilt (BY 1-2 (S)) was used as the template for PCR amplification. A PCR was conducted using an Applied Biosystems 2720 Thermal Cycler (Applied Biosystems, Waltham, MA, USA). PCR reactions were conducted in a volume of 20 μL with the following components: 10 μL of 2× Taq Master Mix (Vazyme, Nanjing, China), 1 μL of template cDNA (100–200 ng/μL), 1 μL of each forward and reverse primer (10 mmol/L), and 7 μL of sterilized double distilled water (ddH_2_O). The thermal cycling conditions were as follows: 95 °C for 3 min (pre-denaturation); 30 cycles of 95 °C for 30 s (denaturation), 55 °C for 30 s (annealing), and 72 °C for 1 min 20 s (extension); and 72 °C for 10 min (final extension). PCR products were separated on a 1% agarose gel in 0.5× trisborate-EDTA buffer and purified using a Star Prep Fast Gel Extraction Kit (GenStar, Beijing, China). The purified PCR products were cloned into the pMD19-T vector (Takara, Beijing, China). The universal primer (pMD19-F/R: CGCCAGGGTTTTCCCAGTCACGAC/CGCCAGGGTTT TCCCAGTCACGAC) was used to detect the PCR products. The positive clones were sent to Sangon Biotech (Shanghai, China) for sequencing.

#### 4.2.3. Bioinformatics Analysis of the Tomato *SlMYB86-like* Gene

The NCBI-ORF Finder was used to determine the ORF of the *SlMYB86-like* gene (accessed on 11 May 2023); the NCBI and SMART online programs were used to analyze the protein domain encoded by the *SlMYB86-like* gene (accessed on 11 May 2023). The *cis*-element motifs of the gene were predicted using the PlantCARE online program (accessed on 31 January 2024). The physicochemical properties and hydrophobicity of the SlMYB86-like protein were predicted using the Expasy ProtParam and Expasy ProtScale online programs, respectively (accessed on 11 May 2023). The secondary structure and tertiary structure of the SlMYB86-like protein were predicted using the SOPMA and SWISS-MODEL online programs (accessed on 11 May 2023). The signal peptides and transmembrane structures of the SlMYB86-like protein were predicted using the SignalP 6.0 and TMHMM Server v.2.0 online programs, respectively (accessed on 11 May 2023). A BLAST search of homologous sequences of SlMYB86-like proteins was conducted in the NCBI database (accessed on 11 May 2023), and MEGA6 software (Version 6.06, Mega Limited, Auckland, New Zealand) was used to compare homologous sequences and construct phylogenetic trees. The PredictProtein online program was used to predict the subcellular localization of the *SlMYB86-like* gene (accessed on 30 January 2024). The specific websites are listed in Table 1.

#### 4.2.4. qRT-PCR Analysis of *SlMYB86-like* in Different Samples

qRT-PCR primers (q*SlMYB86-like*-F/R: CAAATTGCAGCAAAATTACCGG/CTTA GTGGCTTGTGGGTATTTG) were designed based on the cloned *SlMYB86-like* ORF sequences. The tomato *Actin* (Solyc03g078400) (*SlACTIN*-F/R: CTCTACATACTTGAG AGGTGCC/AGACGAGGAGAAAACATCACAA) gene was used as an internal control; the qRT-PCR was conducted using a StepOne Real-Time PCR System and corresponding software (Applied Biosystems, USA). PCR reactions were conducted in a volume of 20 μL with the following components: 0.4 μL of each forward and reverse primer (10 mmol/L), 10 μL of 2× RealStar Green Fast Mixture (with ROX), 0.5 μL of cDNA, and 8.7 μL of RNase-free water. The thermal cycling conditions were as follows: 95 °C for 2 min (pre-denaturation); 40 cycles of 95 °C for 15 s (denaturation), and 60 °C for 20 s (annealing/extension); and a dissolution curve was generated automatically by the instrument. Three biological replicates were performed for each sample. The 2^−ΔΔCt^ method was used to determine the relative expression of *SlMYB86-like* [48].

#### 4.2.5. VIGS of the Tomato *SlMYB86-like* Gene

Primers were designed using the cloned ORF sequence of *SlMYB86-like* (*VSlMYB86-like*-F/R: CCGGAATTCAATACCCACAAGCCACTAAG/CGCGGATCCTTGGAACTCATACAGAAACA). The PCR amplification procedure, PCR reaction system, and thermal cycling conditions were the same as those described in Section 4.2.2. The pTRV2 plasmid and a 252-bp PCR-purified product were digested using the *EcoR* I and *BamH* I enzymes at 37 °C for 2 h. This was followed by enzyme inactivation at 70 °C for 15 min, and T_4_ ligase (Takara, Beijing, China) was applied to recombine the pTRV2 vector and PCR-purified product. The recombination system (10 μL) comprising various components (0.5 μL of T_4_ DNA ligase (350 U/μL), 2 μL of 10× T_4_ DNA ligase buffer, 5 μL of PCR-purified product, 2 μL of pTRV2 vector, and 0.5 μL of ddH_2_O) was left overnight at 16 °C. The next day, the recombination products were transformed into *E. coli* cells DH5α (Guangzhou, China) using the heat-shock method, and the TRV::SlMYB86-like recombinant vector was constructed. The pTRV2 Empty vector (TRV::Empty) and recombinant vector (TRV::SlMYB86-like) were transformed into *Agrobacterium* GV3101 (Guangzhou, China), and the pTRV-1 strain was compared with the TRV:: Empty and TRV::SlMYB86-like strain 1:1 (*v*/*v*), which were mixed and injected into the leaves of Hm 2-2 (R). After 2 d, *R. solanacearum* was inoculated using root-cut irrigation, and its phenotype was observed, photographed, and sampled 5 to 7 days later. Details on specific steps are provided by Chen et al. [49]. Total RNA was extracted from the tomato samples, and qRT-PCR was used to determine the expression of *SlMYB86-like*. The reaction system and thermal cycling conditions were the same as those described in Section 4.2.4.

## 5. Conclusions

The tomato is an economically important crop; however, various biotic stresses, including *R. solanacearum*, TYLCV, and *Botrytis cinerea*, significantly hinder its growth and production. MYB TFs play a key role in regulating the responses of plants to pathogens; however, the role that these TFs play in the response to *R. solanacearum* stress has not yet been clarified. The full-length coding sequence of the *SlMYB86-like* gene was cloned from a tomato, and its structure and function were analyzed. *SlMYB86-like* was most highly expressed in tomato leaves, followed by the stems and roots. The expression of *SlMYB86-like* can be induced by *R. solanacearum*, SA, and MeJA. VIGS assays demonstrated that *SlMYB86-like* promotes the resistance of tomatoes to bacterial wilt. The *cis*-acting element motif prediction of the *SlMYB86-like* promoter showed that *SlMYB86-like* might bind to the promoter to regulate the expression levels of target genes; however, additional studies are needed to confirm this prediction. Our findings will aid future studies aimed at clarifying the molecular mechanisms by which *SlMYB86-like* regulates the resistance of the tomato to bacterial wilt; such studies are necessary for enhancing the production and yield of rapeseed under pathogen stress.

## Figures and Tables

**Table 1:** Websites used in the study.

Tools	Website
NCBI-ORF	https://www.ncbi.nlm.nih.gov/orffinder/ (accessed on 11 May 2023)
NCBI-PROGRAM	https://blast.ncbi.nlm.nih.gov/Blast.cgi?PROGRAM=blastp&PAGE_TYPE=BlastSearch&LINK_LOC=blasthome (accessed on 11 May 2023)
SMART	https://smart.embl.de/smart/set_mode.cgi?NORMAL=1 (accessed on 11 May 2023)
PlantCARE	https://bioinformatics.psb.ugent.be/webtools/plantcare/html/ (accessed on 31 January 2024)
EXPASY-ProtParam	https://web.expasy.org/protparam/ (accessed on 11 May 2023)
EXPASY-ProtScale	https://web.expasy.org/protscale (accessed on 11 May 2023)
SOPMA	https://npsa-prabi.ibcp.fr/cgi-bin/npsa_automat.pl?page=%20npsa_sopma.html (accessed on 11 May 2023)
SWISS-MODEL	https://swissmodel.expasy.org/ (accessed on 11 May 2023)
SIGNALP 6.0	https://services.healthtech.dtu.dk/service.php?SignalP (accessed on 11 May 2023)
TMHMM Server v.2.0	https://services.healthtech.dtu.dk/service.php?TMHMM-2.0 (accessed on 11 May 2023)
PredictProtein	https://predictprotein.org/home (accessed on 30 January 2024)

## Data Availability

Data are contained within the article.

## Supplementary Materials

The following supporting information can be downloaded at: https://www.mdpi.com/article/10.3390/plants13040488/s1, Table S1: Main regulatory motifs found within the promoter sequence of *SlMYB86-like* in tomato.
