# Supplementary material for: Cloning, Expression, and Functional Analysis of the MYB Transcription Factor SlMYB86-like in Tomato
Source: Plants (Basel). 2024 Feb 8;13(4):488. doi: 10.3390/plants13040488 (PMC10893056; doi:10.3390/plants13040488)
Supplement: Supplementary file 1 [file plants-13-00488-s001.zip › plants-2825797-supplementary.pdf]

Table S1. Main regulatory motifs found within the promoter sequence of *SlMYB86-like* in tomato.

Factor or site name	Site	Signal sequence	Function
ABRE	1389(+)	ACGTG	cis-acting element involved in the abscisic acid responsiveness
Box 4	1810(-)	ATTAAT	part of a conserved DNA module involved in light responsiveness
Box III	748(+)	atCATTTTCACt	protein binding site
CAAT-box	165(+), 393(+), 499(+), 507(+), 520(-), 541(-),847(+), 898(+), 906(-), 1012(+), 1135(+), 1241(-), 1257(-), 1296(+), 1415(+), 1624(-),1644(+), 1717(-), 1744(+), 1873(+), 1947(+), 1965(+)	CAAT	common cis-acting element in promoter and enhancer regions
G-box	1371(-), 1388(+)	CACGAC,TACGTG	cis-acting regulatory element involved in light responsiveness
GATA-motif	1084(-)	GATAGGA	part of a light responsive element
GT1-motif	358(+)	GGTTAA	light responsive element
I-box	1516(+)	AAGATAAGGCT	part of a light responsive element
P-box	1970(-)	CCTTTTG	gibberellin-responsive element
TATA-box	145(-), 223(-), 776(-), 1385(-), 1443(-), 1710(-), 1935(-),	TATAA	core promoter element around -30 of transcription start
TC-rich repeats	467(+)	GTTTTCTTAC	cis-acting element involved in defense and stress responsiveness
TCA-element	1092(-)	CCATCTTTTT	cis-acting element involved in salicylic acid responsiveness
TCT-motif	471(+), 1833(-)	TCTTAC	part of a light responsive element
TGA-element	1496(-)	AACGAC	auxin-responsive element
WUN-motif	266(+), 1021(+)	AAATTTCTT	wound-responsive element
circadian	1947(+)	CAAAGATATC	cis-acting regulatory element involved in circadian control
MYB-like sequence	183(+), 1508(-)	TAACCA	core binding site of MYB factor domain
